# Combining a Sinus Augmentation With Tooth Transplantation When Replacing a Missing Premolar in a Young Patient

**DOI:** 10.1155/crid/8464941

**Published:** 2025-09-12

**Authors:** Jessica Juslin, Tuija Teerijoki-Oksa, Päivi Jääsaari, Tia Kurki, Hanna Thorén

**Affiliations:** ^1^Department of Oral and Maxillofacial Surgery, University of Turku, Turku, Finland; ^2^Department of Oral and Maxillofacial Diseases, Turku University Hospital, Turku, Finland

## Abstract

This case report reports how tooth transplantation and a graft-free sinus lift were combined due to insufficient bone volume at the recipient site. An unerupted wisdom tooth was autotransplanted to replace a missing upper premolar. First, the donor tooth was exposed and gently mobilized. At the recipient site, there was a thin layer of bone under the maxillary sinus. The lateral window technique was used in the sinus lift. After elevating the mucous membrane of the sinus floor, the bone was prepared to match the measures of the donor tooth. The mucous membrane was slightly perforated. The donor tooth was moved to the recipient site, and the buccal root partially lacked bony coverage. The transplanted tooth was fixated with sutures. At the 4-year follow-up control, the transplanted tooth was fully erupted without any clinical or radiographical signs of pathology. If the periodontal ligament of the transplanted donor tooth is delicately handled, it can preserve and facilitate the growth of the alveolar bone. During follow-up, it was obvious that new bone had formed around the roots of the donor tooth. It is possible to successfully transplant a developing tooth in the maxillary premolar region, although the initial bone volume is insufficient.

## 1. Introduction

Tooth transplantation is a well-established treatment procedure when replacing missing teeth among growing patients when orthodontic space closure is contraindicated [1]. If the periodontal ligament of the transplanted donor tooth is delicately handled, it can restore missing bony material [2].

Extension of the maxillary sinuses into the alveolar ridge, together with loss of bone height, may prevent placement of a tooth transplant in the posterior maxillary area due to insufficient bone support. In a graft-free sinus augmentation, bone volume can be increased by suturing the Schneiderian membrane to the lateral wall, creating a void space, and coagulated blood acts as a scaffold for new bone to form [3].

This case report describes the combination of tooth transplantation with a one-stage graft-free sinus augmentation.

## 2. Case Presentation

A nearly 13-year-old boy was referred from the public health care center to Turku University Hospital due to several congenitally missing teeth. The patient reported no subjective problems regarding his appearance or bite. The family history indicated that hypodontia is common in the patient's family.

**Figure 1:**
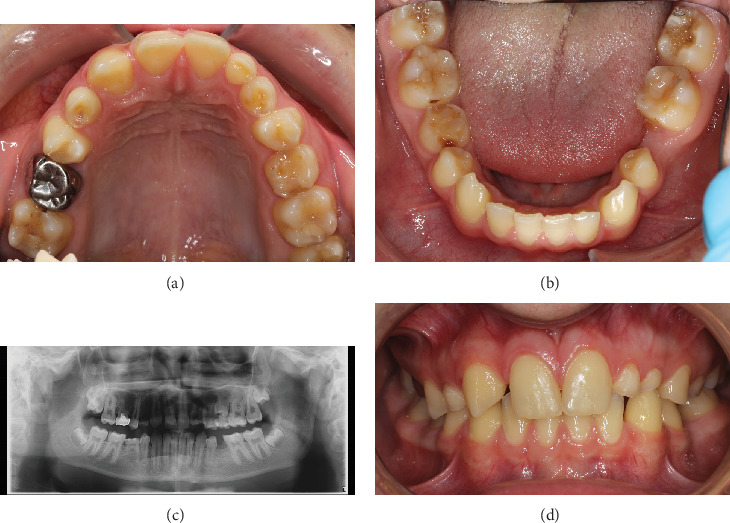
Intraoral photographs and x-ray from a control visit in 2018 resemble the initial state. (a) Multiple retained deciduous teeth in the maxilla. Wear on the deciduous teeth is evident. (b) A persisting deciduous molar and a large gap in the region of the missing left second premolar in the mandible. (c) An orthopantomogram reveals the gravity of the overall situation. Four permanent teeth were missing in the maxilla and two in the mandible. (d) On the right side, the canine has erupted next to the central incisor. The lower canines have continued eruption due to missing antagonists.

In June 2017, an orthodontist and a prosthodontist clinically evaluated the patient. There was no facial asymmetry, his profile was slightly convex, his lip closure was normal, and there was no occlusal tilting. The dentition was in late mixed dentition. There were multiple persisting deciduous teeth (Figure 1a,b), and the panoramic radiograph showed that six permanent teeth were missing (Figure 1c).

He had Class I molar relation on both sides, with an overjet of 3 mm and an overbite of 4 mm. Dental midlines coincided with each other but deviated from the facial midline by 1.5 mm (Figure 1d). There was no functional shift. The upper right second deciduous molar had a preformed metal crown. The persisting deciduous lateral and canines had a lot of wear, and the right canine was mobile and the root was fully resorbed. The right deciduous lower second molar was in infraocclusion, and there was a previous composite build-up. The lower canines and the right first premolar had continued eruption and had now been elongated because of missing antagonists. It was planned to wait with further planning until the eruption of all the permanent teeth.

**Figure 2:**
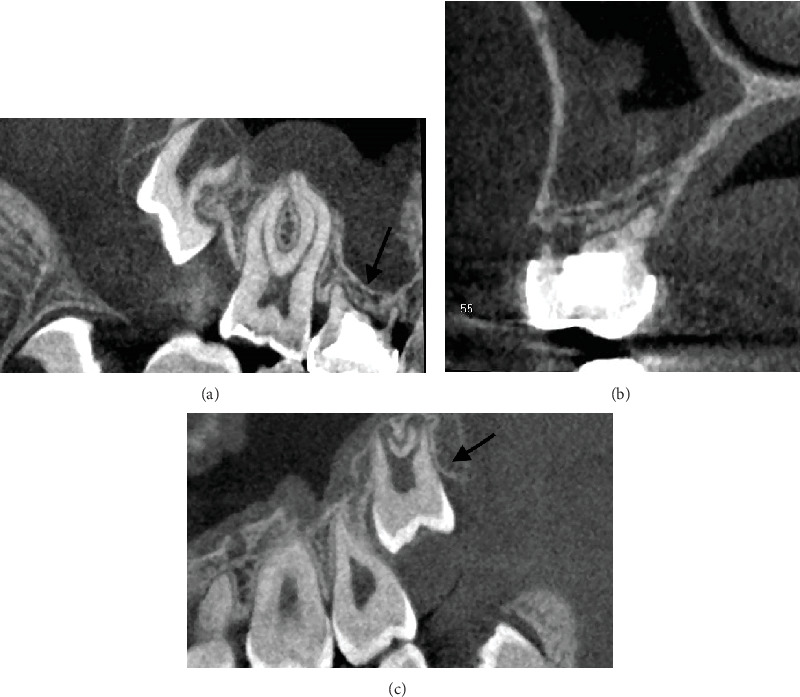
Cone beam tomography was taken to fully understand the condition of the alveolar bone in the recipient site and to evaluate if the third molar was a suitable donor tooth. (a) From the coronal view, the dimensions of the deciduous molar could be measured. (b) The sagittal view revealed how thin the maxillary bone is between the oral cavity and the sinus. (c) The roots of the third molar had developed to three-quarters of their length. The width of the crown exceeded the width of the persisting deciduous tooth in the recipient area.

In November 2019, two surgeons were also consulted. It was planned to autotransplant the left maxillary third molar tooth to replace the missing right second premolar, although the maxillary bone was 2.5 mm thick between the sinus floor and the oral cavity (Figure 2a,b). The root length of the donor tooth was 7.5 mm, and its crown width exceeded the crown width of the deciduous tooth (Figure 2c). Due to the thin layer of bone, it was planned to combine tooth transplantation with a one-stage sinus augmentation. The preprosthetic orthodontic plan and the prosthetic rehabilitation plan would be made in more detail later.

In November 2020, the surgical procedure took place. First, the donor tooth was exposed and gently mobilized, and then it was left in place until the recipient site was fully prepared. Next, a marginal and distobuccal incision was made from the right deciduous second molar to the right third molar tooth, and the periosteal flap was elevated. The deciduous second molar in the recipient area was extracted. Some buccal and apical osteotomies were made in the recipient site to expose the mucous membrane of the floor of the sinus. The lateral window technique was used in the sinus augmentation procedure. The mucous membrane was gently lifted in a sinus-lift kind of manner. The recipient site was then further prepared to match the measurement of the donor tooth, which caused a slight perforation in the mucous membrane. The donor tooth was then extracted and carefully moved to the recipient site. The buccopalatal width of the roots exceeded the width of the alveolar bone in the recipient site, resulting in the buccal root having a partial marginal lack of bony coverage. The transplanted tooth was fixated in place using Vicryl 4-0 and Safil 5-0 sutures.

The patient's instructions included a soft food diet for 2 weeks. Subscribed medications were amoxicillin for 7 days, opening nose spray for 2 weeks, and pseudoephedrine and antihistamine to minimize mucosal swelling for 2 weeks. Painkillers were instructed to be taken when needed. Chlorhexidine mouth rinse was recommended for 2 weeks, twice a day.

A follow-up control occurred following the Turku University Hospital's normal autotransplantation protocol: after 2 weeks, 1 month, 3 months, 6 months, and 12 months. A clinical and radiological examination took place during every visit.

**Figure 3:**
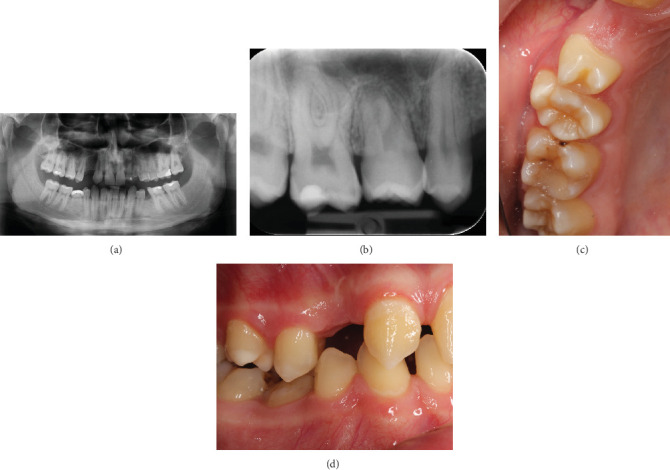
At the 1-year follow-up visit. From (a) the orthopantomogram and (b) the periapical radiograph, we see that the root formation has continued, pulpal obliteration is evident, and there is no sign of pathology. (c) Occlusal view. (d) Side view.

After 1 year, the transplanted tooth continued to erupt, and its palatal cusp was in contact with the antagonist. Normal percussion sound, normal color, and normal mobility of the transplanted tooth were confirmed. The bone volume below the maxillary sinus had increased after the surgical procedure. Pulpal obliteration and continuation of root development were evident from the x-ray (Figures 3a, 3b, 3c, and 3d).

**Figure 4:**
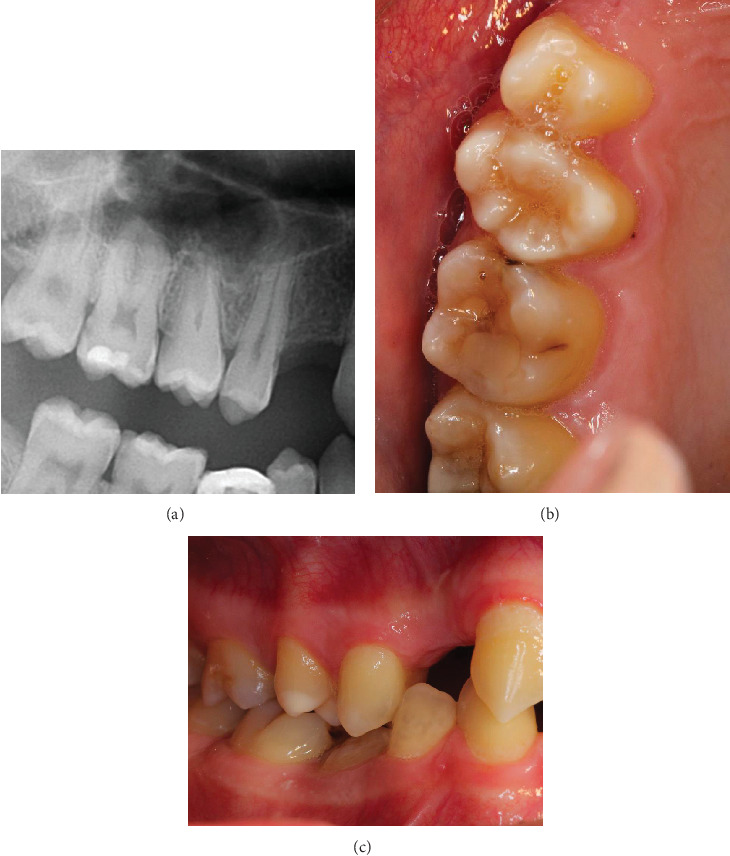
At the 4-year follow-up visit. (a) The bone volume below the maxillary sinus had increased after the sinus lift and transplantation procedure. (b, c) Intraoral photographs show a normal color of the tooth and that the transplanted tooth is now fully in contact with the antagonist.

Four years after the procedure, the normal percussion sound, color, and mobility of the transplanted tooth were still confirmed. The transplanted tooth was fully in contact with the antagonist, and no evidence of pathology was noticed (Figures 4a, 4b, and 4c).

In the future, orthodontic treatment with fixed appliances will be planned for both dental arches, with consideration given to subsequent implant-supported prosthetic rehabilitation. In the mandibular arch, sufficient space is created for an implant to replace the second premolar. Additionally, intrusion of the mandibular canines and closure of existing diastemas are indicated. In the maxillary arch, the treatment plan includes placement of a single implant on each side. An implant-supported bridge will replace the missing lateral and canine teeth.

## 3. Discussion

This case report describes how a graft-free sinus augmentation was combined with tooth transplantation. Lundgren et al. were one of the first to insert endosseous implants in a void space created by graft-free sinus augmentation. It was conceived that bone formation and healing in the void space require the differentiation of osteogenic cells into osteoblasts that deposit collagenous extracellular matrix for mineralization. These mesenchymal stem cells migrate from the bone marrow in the adjacent alveolar bone or from the periosteum of the lifted periosteum. It is suggested that the elevation of the sinus membrane facilitates the formation of a stable blood clot, which then ossifies secondarily [4]. Nedir et al. observed that bone gain slightly continued increasing over the 2 years, thus differing from grafting materials that shrink over time [5]. The minimal remaining alveolar crest height of 3 mm must be present for sufficient primary implant stability [6]. In the occurring patient case, the maxillary bone was 2.5 mm thick between the sinus floor and the oral cavity before sinus augmentation.

The most reported complication for sinus augmentation procedures is the Schneiderian membrane perforation, with an incidence rate of 15%–25%. Chronic rhinosinusitis and hemorrhage are also some possible complications [7]. To minimize complications, the patient was prescribed antibiotics, opening nose spray, pseudoephedrine, and antihistamine. Despite a small perforation in the sinus membrane, the operated area remained infection-free during follow-up.

Autotransplantation is a good treatment option for growing patients to replace missing teeth since they are more likely to have developing teeth with open apices that have a better chance for pulpal healing compared to teeth with a closed apex [1]. The overall success of transplanted teeth is high. When comparing different donor tooth types, teeth with simpler root morphology are better options for a transplant [8].

Delicate handling during the procedure includes minimal extra-alveolar time and minimal fitting attempts of the donor tooth. Compression and injury of the periodontal ligament can predispose root resorption. Multiple fitting attempts of the donor tooth can further increase extra-alveolar time and the risk of trauma to the periodontal ligament. If the periodontal ligament of the transplanted donor tooth is delicately handled, it can preserve and facilitate the growth of the alveolar bone [2]. Although the buccal root of the transplanted tooth partially lacked bony coverage immediately after the operation, it was evident during follow-up that new bone had formed around the root.

Surgical success increases with increasing experience of the team of professionals. Careful selection of patients, meticulous patient information, close collaboration between the orthodontist and surgeon, and standardized treatment and follow-up protocols are of utmost importance [9]. The increased experience of the team was possibly one of the most important factors in this successful patient case.

To our best knowledge, only one similar case has been reported in 2011. Pang et al. combined transalveolar transplantation with sinus augmentation bone graft. An impacted premolar was transplanted to a better position in its site. One cubic centimeter of allogenic bone material (OrthoBlast II; Isotis, Irvine, CA, United States) was used, and it was grafted into the prepared recipient site. The authors believed that the graft material interrupted the healing of the donor tooth since the pulp became necrotic and root canal treatment started 24 days after transplantation [10]. In this current case, no graft material was used, so there was nothing that would interfere with the healing process of the donor tooth. We believe that if the periodontal ligament of the donor tooth is delicately handled, it can preserve and facilitate the growth of the alveolar bone also in situations where the remaining alveolar crest is very thin.

## 4. Conclusion

An experienced team can plan an autotransplantation procedure, although the circumstances are inconvenient. A tooth can be transplanted successfully in the maxilla when the bone volume is limited in the recipient area.

## Data Availability

Data sharing does not apply to this article as no datasets were generated or analyzed during the current study.
